# Assessment and impact of dose escalation on anti-drug antibodies in Fabry disease

**DOI:** 10.3389/fimmu.2022.1024963

**Published:** 2022-12-09

**Authors:** Malte Lenders, Eva Brand

**Affiliations:** Department of Internal Medicine D, Interdisciplinary Fabry Center (IFAZ), University Hospital Muenster, Muenster, Germany

**Keywords:** anti-drug antibodies (ADA), enzyme replacement therapy, dose escalation, Fabry disease (FD), Lyso-Gb3

## Abstract

Enzyme replacement therapy (ERT) with recombinant α-galactosidase A (AGAL) can lead to the formation of neutralizing anti-drug antibodies (ADA), which significantly limit treatment efficacy in patients with Fabry disease (FD). The effects of dose escalation on ADA titer and plasma globotriaosylsphingosine (lyso-Gb_3_) level are unknown. We screened 250 FD patients (200 males, 50 females) under ERT for ADAs and assessed the impact of an approved dose escalation in affected patients, focusing on ADA titers and plasma lyso-Gb_3_. ADA-positive patients were identified by serum-mediated inhibition assays, followed by titration assays to determine the individual inhibitory capacities of ADAs against agalsidase-alfa and agalsidase-beta. 70 (35%) of the male patients were ADA-positive, with a mean inhibitory capacity of 83.5 ± 113.7mg AGAL. Although patients receiving agalsidase-beta showed higher inhibitory capacities (84.7 ± 34.7mg) than patients under agalsidase-alfa (60.3 ± 126.7mg, p<0.001), the “theoretical deficit” to the infused dose was lower in patients receiving agalsidase-beta. In seven patients receiving agalsidase-alfa (0.2 mg/kg) ADAs were saturable by switching patients to agalsidase-beta (1.0 mg/kg). The switch resulted in increasing ADA titers within the first months. In 2 out of 7 (28.6%) therapy switchers, dose escalation could lead to durable ADA saturation. Independent of an increase in ADA titers, lyso-Gb_3_ levels decrease and cardiac and renal parameters remained stable after dose escalation. Dose escalation results in a heterogeneous, unpredictable ADA response, with more than a quarter of all treatment switchers succeeding in ADA saturation. Longitudinal ADA measurements are required to assess the individual risk of affected patients.

## Introduction

Fabry disease (FD; OMIM #301500) is an X-linked (Xq22.1) inborn error of glycosphingolipid catabolism resulting from deficient α-galactosidase A activity (AGAL; 300644) due to mutations in the *GLA* gene. FD-specific manifestations mainly result from the differential systemic accumulation of globotriaoslyceramide (Gb_3_) in the cellular lysosomes (1). FD is a multisystemic disease and onset of first symptoms (acroparesthesia, angiokeratoma, abdominal pain, Cornea verticillata and hypo- or hyperhidrosis) in affected hemizygous males with low or absent enzymatic AGAL activity starts in early childhood (2–4). The persistent accumulation of Gb_3_ in cells of different tissues leads to an early onset of stroke, heart or renal failure, and cardiac arrhythmias resulting in a severely reduced life expectancy.

Enzyme replacement therapy (ERT) with recombinant AGAL (i.e. agalsidase-alfa/-beta) was demonstrated to be effective in reducing intracellular Gb_3_ and lyso-Gb_3_ levels resulting in an improvement of clinical outcomes (4–6). However, intravenous infusion of ERT may cause anti-drug antibody (ADA) formation with neutralizing effects on ERT especially in male FD patients. A positive antibody status is associated with an impaired therapeutic effect on Gb3 reduction and consequently impaired clinical outcome of affected patients (7–10). Inhibitory ADAs in FD are mainly IgG1 and IgG4 (4, 10) and first antibodies could be expected after up to 6 weeks from ERT initiation. Current literature demonstrates a plateau for lyso-Gb_3_ after 6 to 12 months after ERT initiation (at a constant dosage) and a peak for inhibitory ADAs can be expected within the same time-period. However, since time-periods for titer and lyso-Gb_3_ measurements were so far not sensitive enough (i.e. every two weeks), additional studies are required addressing this topic more precisely.

FD patients with neutralizing ADAs might benefit from immune modulatory protocols to reduce titers (11), but aggressive therapeutic strategies as applied for other lysosomal diseases (12, 13) would be accompanied by (severe) side effects and are therefore not yet implemented in clinical routine in FD patients. Recent studies demonstrated that ADA titers can be saturated during infusions (14). As an alternative approach patients with moderate ADA titers might benefit from an approved dose escalation (i.e. switch from agalsidase-alfa [0.2 mg/kg body weight] to agalsidase-beta [1.0 mg/kg body weight]) to saturate all free ADAs. It is still unclear whether dose escalation could also trigger further antibody production in the long term. However, some studies demonstrate that even in patients with ADAs a higher dose of infused enzyme has a beneficial effect on the biochemical response (i.e. lyso-Gb_3_) as well as disease progression (14–16).

Here, we screened for patients with ADAs in a cohort of 200 males and 50 females with genetically confirmed FD and analyzed if individual infused enzyme doses are sufficient to saturate free ADAs in affected patients. Furthermore, we analyze whether the neutralizing properties of ADAs against agalsidase-alfa are similar to those against agalsidase-beta and vice versa. As a potential therapeutic approach in patients with moderate ADA titers, we assessed whether an approved dose escalation (switch from agalsidase-alfa [0.2 mg/kg body weight] to agalsidase-beta (1 mg/kg body weight) triggers further ADA formation. Finally, we analyzed the effect of dose escalation on longitudinal outcomes in a subset of patients.

## Material and methods

### Patients’ samples

Pseudonymized serum samples from Fabry patients (200 males, 50 females; including patients from our Fabry center) were sent to our laboratory by various international Fabry centers with the request to test them for the presence of neutralizing ADAs and, if detected, to analyse the resulting inhibitory capacities against agalsidase-alfa or agalsidase-beta. All investigations were performed after approval by the Medical Association of Westphalian-Lippe and the Ethics Committee of the Medical Faculty of the University of Muenster (project no. 2011-347-f, date of report: July 7, 2011) and in accordance with the Declaration of Helsinki. Written informed consent was obtained from all included patients for analysis and publication. Plasma lyso-Gb_3_ levels from dried blood spots (DBS) available from 28 patients with neutralizing antibodies and known treatment status (agalsidase-alfa or agalsidase-beta) were measured by Centogene AG, Rostock, Germany.

Blood sampling for ADA measurement was performed directly before the next infusion, to minimize interferences with infused enzymes.

### Serum-mediated inhibition assays to detect neutralizing ADAs in blood samples

Serum-mediated inhibition assays (7, 9, 17) were used to detect the presence of neutralizing ADAs in serum samples from males and females.

### Purification of total IgGs from human sera for titration assays

Total IgGs from patients’ sera for titer measures were purified by negative selection as described previously using Melon Gel IgG Spin Purification Kit (Thermo Fisher Scientific, Darmstadt, Germany) according to manufacturer’s instructions (14, 16). In brief, 100 µl serum were diluted 1:10 with Melon Gel buffer, incubated with 100 µl settled Melon Gel, and inverted for 5 min at room temperature. After protein adsorption, total IgGs were separated *via* centrifugation at 12,000 x g for 5 min. BCA (Thermo Fisher Scientific) and SDS-PAGE analysis was performed as reported previously to estimate the purified IgG content and to control the success of purification (14, 16).

### Titration of neutralizing ADAs to assess inhibitory capacities

The amount of enzymes (agalsidase-alfa or agalsidase-beta) required to saturate ADAs in patients’ sera was determined as described previously (14, 16). In short, 5 µg patients’ purified total IgGs were pre-incubated with a serial dilution of AGAL enzymes (agalsidase-alfa or agalsidase-beta) for 10 min at room temperature. To express enzyme inhibition in percent, residual AGAL activities were normalized against inhibition-negative controls. Enzyme inhibition was plotted against the amount of agalsidase and saturation was defined as the amount of enzyme required to reduce the neutralizing capacity of 5 µg patients’ total IgG below the AGAL neutralizing threshold of 10% (background threshold) (14, 16).

### Statistical analyses

Unless otherwise indicated, measures were performed in triplets. Categorical data are expressed as numbers and relative frequencies as percentages. Two-tailed Student’s t tests or one-way analysis of variance (ANOVA) with correction for multiple testing were used for statistical analysis. Statistical significance was considered at a two-sided p<0.05. GraphPad PRISM V5.0 software (GraphPad Software Inc, La Jolla, California) was used for appropriate statistical analyses and visualization.

## Results

### Identification of ADA-positive patients

**Figure 1:**
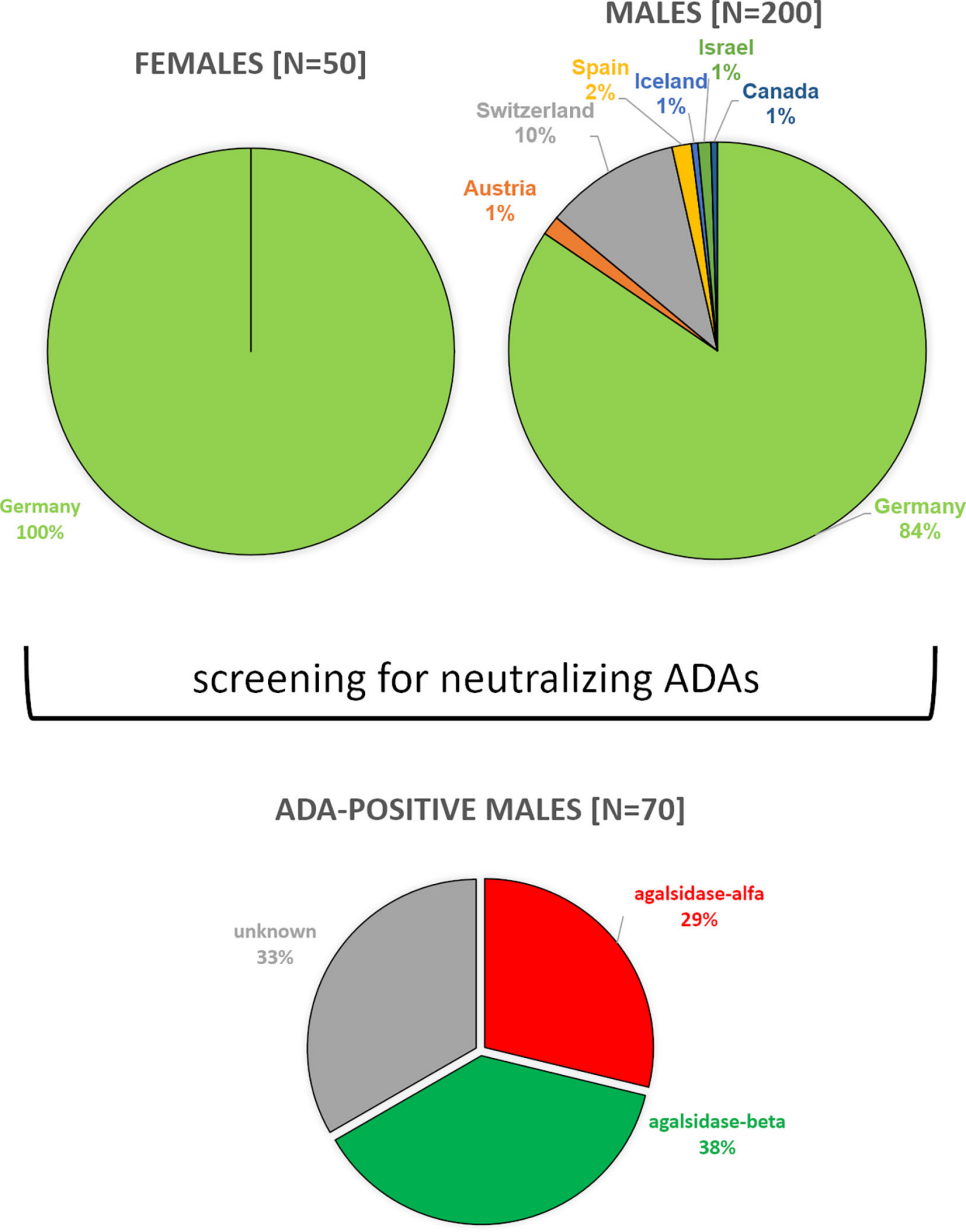
Overview of the study population.

**Figure 2:**
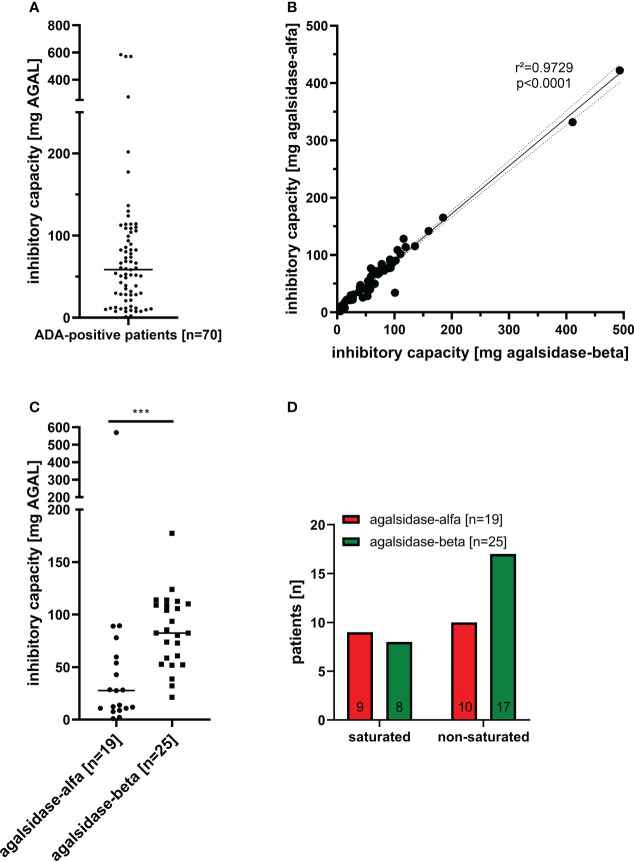
Inhibitory capacities of neutralizing anti-drug antibodies in male FD patients under enzyme replacement therapy. **(A)** Overall inhibitory capacities in all ADA positive patients (marked median value). **(B)** Correlation of inhibitory capacities for agalsidase-alfa and agalsidase-beta. **(C)** Inhibitory capacities (median values) in patients treated with agalsidase-alfa or agalsidase-beta. **(D)** Saturation status (saturated, unsaturated) under agalsidase-alfa or agalsidase-beta. AGAL: α-galactosidase A ***p<0.0001.

Between 08/2019 and 03/2022, we screened serum samples from 200 male patients and 50 female patients with FD under enzyme replacement therapy (agalsidase-alfa or agalsidase-beta) for the presence of neutralizing ADAs to identify patients at risk. Serum samples were provided mainly from Germany (males: n=169; females: n=50), but also from Austria (males: n=3), Switzerland (males: n=21), Canada (males: n=1), Israel (males: n=2), Iceland (males: n=1), and Spain (males: n=3) (**Figure 1**). Using serum mediated-inhibition assays (7, 9) for screening, we identified 70 (35%) male patients as positive and all tested females as negative for neutralizing ADAs. Information on the (ADA-positive) patients, including age, current ERT treatment (agalsidase-alfa or agalsidase-beta), and plasma lyso-Gb3 levels, was available only for a subset of patients: Mean age was 42 ± 14 years (n=44). Current treatment status was known from 44 patients (19 patients receiving agalsidase-alfa, 25 patients receiving agalsidase-beta). Plasma lyso-Gb_3_ (37.7 ± 23.4 ng/ml) and genotypes were known from 28 patients of these 44 patients. Only 7 (25.0%) of these 28 patients carried a missense mutations (2*p.I91T, p.C94S, p.C202Y, p.S247P, p.L294S, p.R301Q) all other patients carried nonsense mutations. The inhibitory capacities of ADAs were measured in all ADA-positive men, reflecting the amount of infused AGAL required to saturate all free antibodies during infusion (AGAL excess) (14, 16). The mean inhibitory capacity of ADAs from all patients was 83.5 ± 113.7 mg agalsidase-beta (**Figure 2A**). Of note, the inhibitory capacities against agalsidase-alfa and agalsidase-beta were similar, confirming the previously described cross-reactivity of ADAs in FD against currently approved recombinant AGAL (7) (r²=0.9729; p<0.0001; **Figure 2B**). Next, we compared the inhibitory capacities (and thus ADA titers) between patients receiving agalsidase-alfa and those receiving agalsidase-beta. At the time of ADA measurement (i.e. serum sampling), patients receiving agalsidase-beta showed significantly higher inhibitory capacities (mean: 84.7 ± 34.7 mg) than those receiving agalsidase-alfa (mean: 60.3 ± 126.7 mg, p<0.001; **Figure 2C**). Assuming a standard dose of 14 mg agalsidase-alfa and 70 mg agalsidase-beta for a patient weighting 70 kg, we found no increased risk for unsaturated patients receiving agalsidase-beta (17/25 patients, 68.0%) compared to patients receiving agalsidase-alfa (10/19 patients, 52.6%, p=0.3588; **Figure 2D**). At the appropriately approved standard doses for agalsidase-alfa (14.0 mg) and agalsidase-beta (70.0 mg), ADAs were not saturated on average (agalsidase-alfa: 14.0 mg [received] vs 60.3 ± 126.7 mg [required for saturation], agalsidase-beta: 70.0 mg [received] vs 84.7 ± 34.7 mg [required for saturation]). However, the “theoretical deficit” (to saturate all free ADAs) was markedly lower in patients receiving agalsidase-beta (100%/84.7 mg * 70 mg = 82.6% → -17.4% deficit) than in patients receiving agalsidase-alfa (100%/60.3 mg * 14 mg = 23.2% → -76.8% deficit).

Therefore, although patients under agalsidase-beta had on average a higher inhibitory capacity and thus a higher antibody titer than patients under agalsidase-alfa, these patients lacked less infused enzyme to saturate all free antibodies due to the 5-fold higher dose. This might explain the observed better biochemical response in patients with ADAs receiving agalsidase-beta (14, 15, 18).

### ADA formation in ERT-naïve patients after treatment initiation

**Figure 3:**
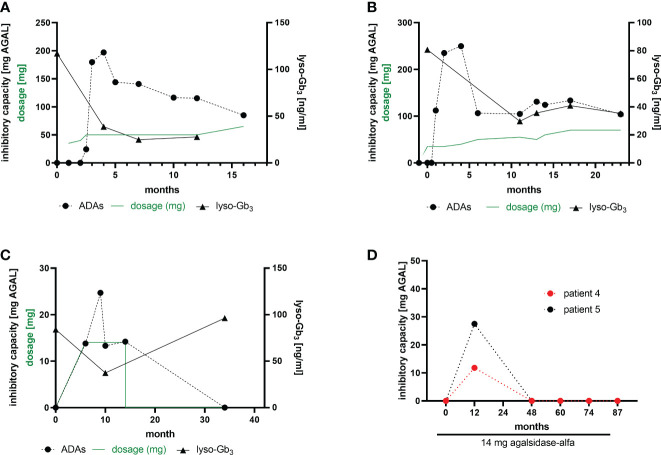
ADA formation in ERT-naïve patients after treatment initiation. **(A, B)** Two patients receiving a stepwise dosage increase after an initial dose of 35 mg agalsidase-beta. **(C)** A patient starting with 14 mg agalsidase-alfa and discontinuing after 14 months of treatment (non-compliance). **(D)** Two patients receiving 14 mg agalsidase-alfa and showing a serum conversion after 12 months of treatment, becoming ADA-negative.

Current data suggest a humoral response (i.e. the formation of antibodies) against infused AGAL within 3 to 6 months after treatment initiation (7, 19). In our cohort, we performed longitudinal measurements in 2 patients, who developed ADAs after ERT initiation with agalsidase-beta after ~6 -10 weeks (**Figures 3A, B**). Both patients showed highest ADA peaks between 3 to 5 months after treatment initiation, followed by a ~40% to 50% reduction after 15 months of treatment. Of note, both patients started with a stepwise dosage increase of agalsidase-beta (starting at 35 mg), which did not prevent ADA formation. Plasma lyso-Gb_3_ levels in these two patients decreased after treatment initiation, but reached a plateau after 6 to 10 months (**Figures 3A, B**). A third patient receiving agalsidase-alfa (14 mg) showed a comparable increase of ADA titers after 6 to 10 months of treatment initiation (**Figure 3C**). However, after 14 months of ERT this patient discontinued treatment for the following 20 months (non-compliance). Interestingly, a recent serum sample showed no ADAs (**Figure 3C**). Most important, in this patient plasma lyso-Gb3 decreased by 50% during the treatment, but re-increased after ERT stop to the initial value (**Figure 3C**). Furthermore, we identified 2 patients on agalsidase-alfa with ADAs, showing a serum conversion followed by a tolerization after 12 months of treatment (**Figure 3D**).

### Impact of dose escalation on ADA titers

**Figure 4:**
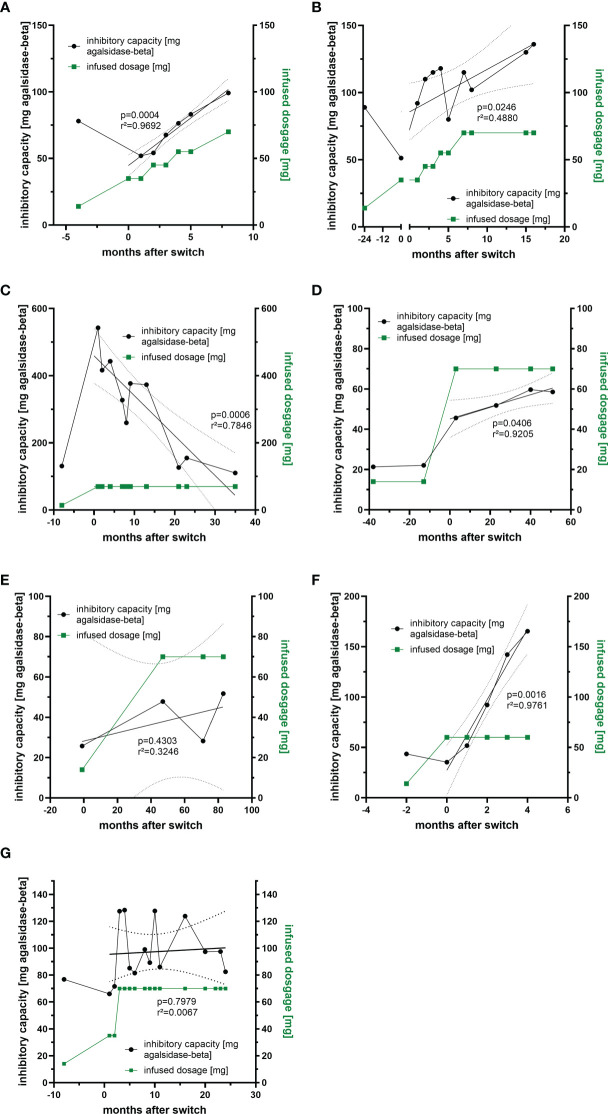
Impact of dosage increase and compound-switch on ADA titers. **(A–C)** Three patients who were switched from agalsidase-alfa to agalsidase-beta, but dosage increase was never sufficient to saturate all free ADAs due to increasing titers. **(D, E)** Two patients who were switched to agalsidase-beta. Although ADA titers increase, infused dosages were sufficient to saturate all free ADAs. **(F)** A patient who was switched to agalsidase-beta, but the dosage increase was sufficient to saturate all free ADAs only in the first two months, as ADA titers increased massively. **(G)** A patient switched to agalsidase-beta showing oscillating ADA titers over time.

Previously we demonstrated that free antibodies can be saturated during infusion, which was associated with improved outcome in affected patients, probably due to the AGAL excess (14). To analyze whether non-saturated patients under agalsidase-alfa might benefit from a 5-fold dosage increase, we measured ADA titers over time in 7 patients who were switched from agalsidase-alfa (0.2 mg/kg body weight) to agalsidase-beta (1.0 mg/kg body weight) (**Figures 4A–G**). A switch from agalsidase-alfa to agalsidase-beta resulted in an increase of ADA titers in all 7 patients within the first months (**Figures 4A–G**). However, the long-term response was more heterogeneous. In detail, four patients experienced a marked increase in ADA titers over time, causing three of them to revert to the unsaturated state despite a 5-fold increase in dose (**Figures 4A-F**). In one patient, after an initial massive increase, the ADA titer decreased significantly over time to the baseline value before the switch (**Figure 4C**). In addition, one patient showed only a slight initial ADA increase after switch, resulting in a saturation of free ADAs (**Figure 4E**). The seventh patient presented with an initial massive increase of ADA titers, too (**Figure 4G**). However, ADA titers oscillated between the initial titer and a peak value over time. Thus, a dose-escalation was successful (in terms of saturation of free ADAs) in 2 (28.6%) of the 7 switched patients. Of note, all switched patients tolerated the dose escalation well and did not report increased frequencies of infusion-associated reactions after the switch.

### Impact of dose escalation on FD-typical cardiac and renal manifestations

**Figure 5:**
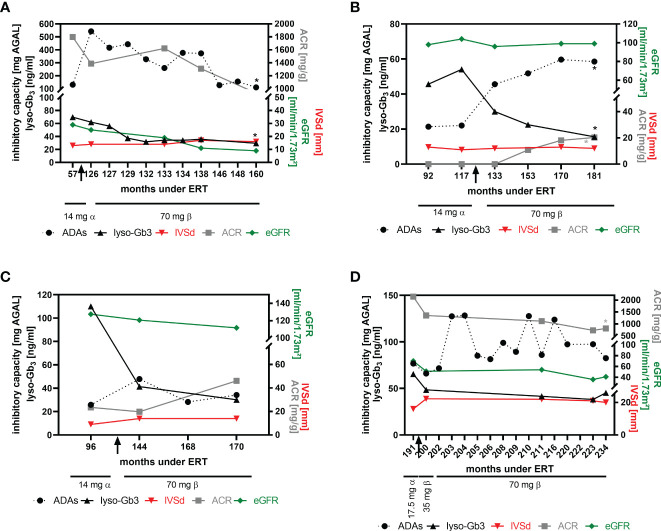
Impact of dose escalation on cardiac interventricular septum thickness and renal function. **(A)** Patient 1, **(B)** patient 2, **(C)** patient 3, **(D)** patient 4. Significant changes (p<0.05) over time are marked by an asterisk (*) in their respective colours. ACR, albumin-to-creatinine ratio, ADAs, anti-drug antibodies, eGFR, estimated glomerular filtration rate, IVSd, interventricular septum thickness at end diastole, lyso-Gb_3_, globotriaosylsphingosine.

Preliminary data from our previous studies suggested that patients with high ADA titers might benefit from a dose-escalation (switch from agalsidase-alfa to agalsidase-beta) at least biochemically (16, 18). To analyze the impact of a dose escalation on FD-typical cardiac and renal manifestations, we were able to follow-up at least 4 patients after the switch more in detail (**Figure 5**). In two patients (patient 1 and patient 2), the ADA titer increased after the switch (**Figures 5A, B**). While the titer in patient 1 steadily decreased during the 34-month follow-up period (**Figure 5A**), the ADA titer in patient 2 increased significantly over time and reached a plateau after ~36 months (**Figure 5B**). ADA titers in patients 3 and 4 remained stable overall (**Figures 5C, D**), although titers in patient 4 showed some oscillation (**Figure 5D**). Lyso-Gb_3_ levels decreased in patients 1, 2 and 3 and remained stable in patient 4 over time (**Figures 5A–D**). All 4 patients presented with a stable interventricular septum thickness and stable eGFR values. Albumin-creatinine ratio was stable in patients 1 and 3 (**Figures 5A, C**), slightly increased in patient 2 (**Figure 5B**, within normal range, without microalbuminuria at the end of observation) and significantly decreased in patient 4 (**Figure 5D**).

## Discussion

The formation of neutralizing ADAs against infused recombinant agalsidase in male FD patients significantly limits the therapeutic efficacy of ERT. Therefore, it is clinically important to identify affected patients at-risk. Here, we analyzed the general presence of neutralizing ADAs in a large cohort of FD patients treated with agalsidase-alfa or agalsidase-beta. We also performed longitudinal measurements to characterize ADA formation in newly treated patients and monitored ADA titers in patients who were switched from agalsidase-alfa (0.2 mg/kg body weight) to agalsidase-beta (1.0 mg/kg body weight) to assess the potential impact of dose escalation on ADA titers over time.

In short, our main findings were: 1) 70 (35%) of all tested male patients under ERT were identified as neutralizing ADA-positive, whereas all female patients tested (n=50) were neutralizing ADA-negative. 2) ADAs showed a high cross-reactivity for agalsidase-alfa and agalsidase-beta. 3) The inhibitory capacities (i.e. ADA titers) in patients treated with agalsidase-alfa were lower compared to patients treated with agalsidase-beta, but the risk for unsaturated ADAs during infusions was comparable. 4) In patients newly treated with ERT, ADAs peak at 3 to 6 months, followed by a slight decrease of titers within ongoing treatment. 5) A dose escalation (i.e. switch from agalsidase-alfa to agalsidase-beta) appears to have heterogeneous long-term effects in patients, and seems to trigger an increase of ADA titers after switch, but could lead to durable ADA saturation (in more than a quarter of patients). 6) Independent of an increase in ADA titers, lyso-Gb_3_ levels decrease and cardiac and renal parameters remained stable over time in patients switched from agalsidase-alfa to agalsidase-beta.

Over the past two decades, the deleterious impact of neutralizing ADAs on therapy efficacy in FD became increasingly apparent (19). To overcome this therapeutic dilemma, the identification of patients at risk and either specific immunomodulatory protocols, modified or novel treatment options need to be established. As a first step, we screened 200 male and 50 female patients with FD under ERT for the presence of neutralizing ADAs. Previous studies demonstrated that patients with saturated ADAs during infusions benefit from an AGAL excess, resulting in a better therapeutic outcome over time, suggesting that non-saturated patients might benefit from a dosage increase of infused AGAL (14–16, 18). Therefore, we screened ADA-positive patients for those with ADA titers that could be saturated when therapy was switched to an approved dose of 1.0 mg/kg b.w. agalsidase-beta. We identified 7 patients under agalsidase-alfa to whom this approach applied. Currently, it is discussed whether ADA titers also increase when infused AGAL doses are increased (for example switching from agalsidase-alfa [0.2 mg/kg b.w.] to agalsidase-beta [1.0 mg/kg b.w.]). Our data demonstrate that all patients showed an ADA titer increase directly after a switch to 1.0 mg/kg b.w. agalsidase-beta. However, the long-term response in our patients was very heterogeneous: While ADA titers in three patients showed a continuous massive increase over time resulting in a non-saturated status, two patients (2/7 patients, 28.6%) showed only a slight increase under agalsidase excess after the end of observation with the permanent saturation of ADAs.

In another patient, a massive increase in ADA titers was followed by a steady decline of titers over 36 months of treatment back to pre-switch titers. The seventh patient showed oscillating titers over time. The observed oscillation might be due to the fact that the patient suffers from severe infusion-associated reactions, requiring a potent pre-medication including high dosages of prednisolone prior to infusions. In particular, the use of glucocorticoids might have an impact on ADA formation, as previously demonstrated in patients with kidney and heart transplantation (11). Blood sampling for ADA measurements was performed directly before the next infusion. This allows ADA titers to re-increase and minimizes potential interferences with infused enzymes and thus cannot explain heterogenous effect of dose escalations. Although the ADA response to dose escalation was heterogeneous and probably not all free ADAs were saturated in patients during infusions, all patients benefited from the switch, as plasma lyso-Gb_3_ levels decreased and cardiac and renal parameters remained stable over time. This might be explained in that the “theoretical deficit” after the switch was lower than before the switch, increasing the likelihood that some functional enzyme still reaches its final destination (i.e. lysosomes).

Of note, we identified one patient who showed absent ADAs after ERT stop. Since half-life of IgGs is generally 3 months and without further stimulus by ERT this was not unusual. It will be interesting now, if the referred patient will again produce ADAs if ERT should be continued by activating memory cells.

In conclusion, we successfully measured ADA titers in a large (international) cohort of FD patients. Our data demonstrate that the ADA response is heterogeneous among affected patients, leading to a wide variation in the resulting ADA titers. If more than a quarter of all patients who do not saturate their ADAs under agalsidase-alfa show ADA saturation after switching to agalsidase-beta, this is a clinically valuable success, especially since the cost remains the same when switching drugs. Clinical controls of relevant organs and longitudinal ADA measures are required to assess the individual risk of affected patients.

## Limitations

Serum samples from patients with FD under ERT were sent to our lab to assess the presence of neutralizing ADAs. In most cases only the age and the sex of the patient was provided. Thus, only for a subset of patients detailed patient-specific information were available. Serum samples were from all kind of male FD patients including late-onset, missense and nonsense mutations. Therefore, the observed frequency of 35% for ADAs seems to be representative for representing a typical FD cohort.

## Data availability statement

The original contributions presented in the study are included in the article/supplementary material. Further inquiries can be directed to the corresponding author.

## Ethics statement

The studies involving human participants were reviewed and approved by Medical Association of Westphalian-Lippe and the Ethics Committee of the Medical Faculty of the University of Muenster. The patients/participants provided their written informed consent to participate in this study.

## Author contributions

Both authors designed the study design and concept. ML conducted the experiments and wrote the manuscript. Both authors collected, analyzed and interpreted the data. EB reviewed and edited the manuscript. Both authors provided resources, read and agreed to the published version of the manuscript.
